# Valorization of Wild Apple (*Malus* spp.) By-Products as a Source of Essential Fatty Acids, Tocopherols and Phytosterols with Antimicrobial Activity

**DOI:** 10.3390/plants7040090

**Published:** 2018-10-24

**Authors:** Vitalijs Radenkovs, Jorens Kviesis, Karina Juhnevica-Radenkova, Anda Valdovska, Tõnu Püssa, Maris Klavins, Inese Drudze

**Affiliations:** 1 Processing and Biochemistry Department, Institute of Horticulture, Latvia University of Life Sciences and Technologies, Graudu Str. 1, LV-3701 Dobele, Latvia; karina.juhnevica-radenkova@llu.lv (K.J.-R.); inese.drudze@puresdis.lv (I.D.); 2 Department of Environmental Science, University of Latvia, Jelgavas Str. 1, LV-1004 Riga, Latvia; jorens.kviesis@inbox.lv (J.K.); maris.klavins@lu.lv (M.K.); 3 Faculty of Veterinary Medicine, Latvia University of Life Sciences and Technologies, 8 K. Helmaņa Str., LV-3004 Jelgava, Latvia; Anda.Valdovska@llu.lv; 4 Research Laboratory of Biotechnology, Latvia University of Life Sciences and Technologies, Strazdu Str. 1, LV-3004 Jelgava, Latvia; 5 Chair of Food Hygiene and Veterinary Public Health, Estonian University of Life Sciences, Kreutzwaldi 56/3, 51014 Tartu, Estonia; tonu.pyssa@emu.ee

**Keywords:** *Malus* spp., oil, FAMEs, tocopherols, carotenoids, MIC

## Abstract

The amplified production of fruit as well as burgeoning demand for plant-made food products have resulted in a sharp increase of waste. Currently, millions of tons of by-products are either being discarded or utilized rather ineffectively. However, these by-products may be processed and further incorporated as functional ingredients in making high-value food products with many physiological and biochemical effects. The chemical analysis of pomace oils using gas chromatography-mass spectrometry (GC/MS) and reversed-phase-liquid chromatography coupled with fluorescence detector (RP-HPLC/FLD) systems led to the identification and quantification of 56 individual lipophilic compounds including unsaturated, polyunsaturated and saturated fatty acids, as well as phytosterols and four homologs of tocopherol. The oils recovered from by-products of *Malus* spp. (particularly cv. “Ola”) are rich in fatty acids such as linolenic (57.8%), α-linolenic (54.3%), and oleic (25.5%). The concentration of total tocopherols varied among the *Malus* species and dessert apples investigated, representing the range of 16.8–30.9 mg mL^−1^. The highest content of total tocopherols was found in *M.* Bernu prieks, followed by *M*. cv. “Ola”, and *M*. × *Soulardii* pomace oils. A significantly higher amount of δ-tocopherol was established in the oil of *M*. Bernu prieks, indicating that this species could be utilized as a natural and cheap source of bioactive molecules. β-Sitosterol was the prevalent compound determined in all tested pomace oils with a percentage distribution of 10.3–94.5%. The main triterpene identified in the oils was lupeol, which varied in the range of 0.1–66.3%. A targeted utilization of apple pomace would facilitate management of tons of by-products and benefit the environment and industry.

## 1. Introduction

Domesticated apple (*Malus* × *domestica* Borkh.) is one of the most important fruit crops worldwide. According to FAOSTAT (2018) [1], the world production of apple in the last five decades has increased by 424% from 17.0 million tons in 1961 to 89.3 million tons in 2016. Leading production nations include (in descending order) China, USA, Poland, Turkey, Iran, Italy, Russia, Uzbekistan, and Ukraine. There are hundreds of apple cultivars, but only five currently dominate world production: Fuji, Golden Delicious, Delicious, Granny Smith, and Gala. Currently, dessert apples are processed into many food products including juice (2.1 million liters among the 28 European Union member states (EU-28)) [2], apple sauce, slices (dried, frozen and canned) and cider (sweet and hard) (1.4 million liters in the EU-28) [3]. The increased production of apples as well as burgeoning demand for plant-derived food products have resulted in dramatic increase of waste. The food processing sector contributes the most to food waste—in 2012, this was estimated to be 17 million tons or 19% of a total amount of 87.6 million tons of food waste generated in the EU-28, based on data provided by Fusions EU Project [4]. In large-scale apple juice production, 75% of the apple are utilized for juice, while the remaining 25% are discarded as waste or utilized rather inefficiently [5]. These by-products may be processed and further incorporated as ingredients in making high-value functional food products (cereal bars, cookies, muffins, bread and fermented milk products) with many physiological and biochemical effects [6].

Dessert apple fruits as well as their by-products have been studied in terms of their chemical composition [7,8], along with their positive health effects [8]. However, rather little is known concerning the lipophilic composition of crab apples. Crab apples, also known as wild apples, belong to the genus *Malus* (*Rosaceae* family) and are currently used not only for ornamental purposes, but also are one of the most important source of seeds with an increased concentration of all four homologs of tocopherol (α, β, γ and δ) [9], and hydrophilic antioxidant substances for the food and cosmetic industries [10]. Food industries have been increasingly focused on developing high-quality food products with increased functionality, therefore crab apples have gained an increasing interest in the last decades [8,11]. Crab apples are used in the preparation of jellies, jams, beverages, and wines [12]. Dadwal et al. [12] found that extracts obtained from either pulp or seeds of Himalayan crab apple fruits (*M. baccata*) not only contained moderate concentrations of polyphenols (phloretin and phloridzin), but also fatty acid molecules such as palmitic acid, ethyl palmitate, methyl petroselinate and linolein, which are well recognized for medicinal uses [13]. Moreover, Górnaś et al. [14] reported that the apple seeds recovered from dessert and crab apple are a promising source of oils, which also contain phytosterols, mainly β-sitosterol.

The most common phytosterols in the human diet are β-sitosterol, campesterol, and stigmasterol [15]. In vitro studies suggest that the consumption of foods or supplements enriched with phytosterols may be a partial way to reduce serum low-density lipoprotein (LDL) cholesterol levels by reducing intestinal cholesterol absorption [16]. Phytosterols have also been found to be effective in preventing lipoprotein oxidation in mice fed with high-fat diets, thus showing anticancer effects [17]. Recent studies have reported that the by-products of *Malus* genus have potential to be used in the production of therapeutic substances with a wide application in pharmaceutical and natural cosmetic industries, thus contributing to the reduction of waste generated during apple processing [9,14,18]. The main objective of this study, analysis of the lipophilic constituents from the pomace of *Malus* crab apple, reflects the growing interest in finding new approaches for the recovery of by-products generated during fruit processing.

## 2. Results and Discussion

### 2.1. FAMEs of Crab Apple Pomace Oil

The composition of FA in oils recovered from the pomace of four crab and one dessert apples analyzed using GC-MS are shown in Table 1.

**Figure 1:**
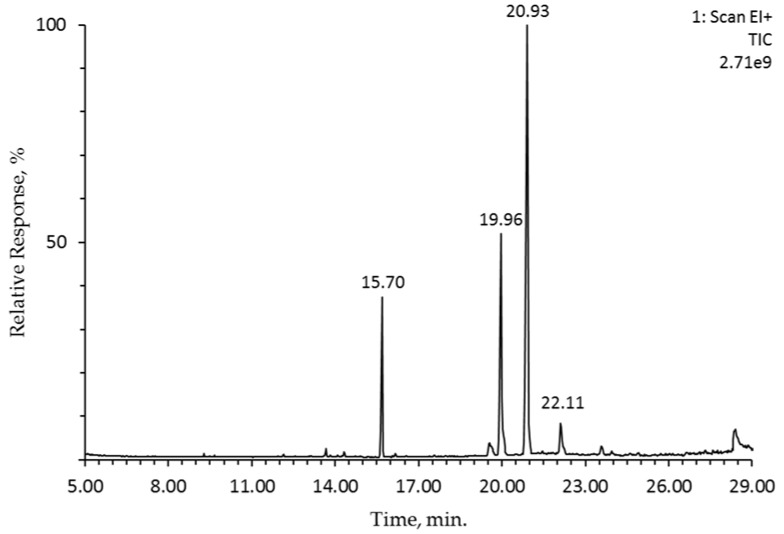
Chromatographic separation of FAMEs profile of crab apple *Malus* Bernu prieks oil using GC/MS: 15.70 min (palmitic acid), 19.96 min (oleic acid), 20.93 min (linoleic acid), and 22.11 min (α-linolenic acid).

Seventeen FAs in the form of methyl esters (FAMEs) were identified and quantified, among which the dominance of unsaturated fatty acids, mostly linoleic acid (from 43.0 to 57.8%), followed by oleic acid (19.4–25.5%), was found (Figure 1). Among the saturated fatty acids, representatives such as palmitic acid (8.7–13.0%) were prevalent among the crab apple oils. Furthermore, similar concentrations of FAs were found in commercial pumpkin and soybean oils [19], where the concentration of linoleic, oleic, and palmitic acid among the FAs amounted to 47.1%, 34.1%, and 10.7% and 50.8%, 24.6%, and 10.2% of the total FAs, respectively. In addition, a similar distribution of FAs, in particular unsaturated fatty acids recovered from apple by-products, which represented ~90% of the total fatty acids has been previously reported [20]. Whereas the opposite has been reported by Pires et al. [21], where the authors using the Soxhlet extraction indicated the dominance of palmitic acid, followed by stearic and linoleic acids (28.94%, 16.4% and 15.8%, respectively) in the oil of *M.* × *domestica* Borkh. cv. Bravo de Esmolfe apples.

Considering the content of minor fatty acid molecules, α-linolenic and arachidic acids were found to be dominant, varying in the range of 1.8–4.7% and 0.7–3.9%, respectively. It must be noted, however, that the amounts of α-linolenic acid reported here are significantly higher than those observed in *Prunus avium* kernel [22], *M. bacata* seed [12], *Vitis* spp. oils [23], and *Viburnum opulus* lipophilic extracts [24].

Only small amounts of less stable and therefore more prone to oxidation FFAs were found (Table 2). Among the FFAs analyzed, linoleic and oleic acid were found to be the dominant compounds in *M*. crab apples, varying in the ranges of 13.3–54.3% and 26.7–32.9%, respectively. Considering the content of oxygenated fatty acids in oils, 9,10-dihydroxystearate was the only representative, and found in small amounts (<0.1% total lipids). This low amount may be due to the relatively high content of Ts responsible for protecting the oil against oxidation [25]. Among the aliphatic alcohols, nonacosan-10-ol was the dominant compound identified in all tested pomace oils, varying in the range of 78.8–90.0%. The presence of nonacosan-10-ol as a typical constituent of the wax and cutin of *Prunus avium* and *Malus* × *domestica* Borkh. cv. “Red Fuji” was also described by Peschel et al. [26] and Dong et al. [27], respectively.

The results of the aforementioned study show that the oils recovered from by-products of *Malus* spp. especially cv. “Ola” are rich in fatty acids such as linoleic and oleic acids. A targeted utilization of apple pomace would facilitate management of dozens of by-products and benefit the environment and industry [6].

### 2.2. Ts of Crab Apple Pomace Oil

**Figure 2:**
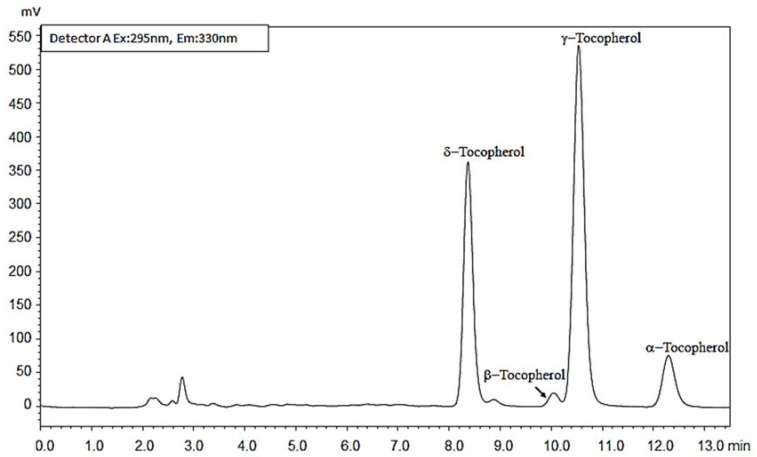
RP-HPLC/FLD chromatogram of tocopherols from *Malus* “Ola” oil separated using a Luna PFP column.

Tocochromanols are a group of the major forms of vitamin E, consisting primarily of four (α, β, γ, and δ) homologs of tocopherol and tocotrienol [9] that are synthesized exclusively by photosynthetic organisms and therefore could be ingested as part of the diet [28]. Vitamin E, is an essential lipid-soluble compound with a unique biological activity [29]. Four Ts (α, β, γ, and δ) were identified and quantified in all crab and dessert apple pomace oils (Figure 2 and Table 1). With the exception of the *M.* Bernu prieks, α-T was the prevailing homolog present in apple pomace oils, with the content in the range of 4.4–13.9 mg mL^−1^ (percentage distribution of 14.1–60.0%). The second abundant homolog, after α-T, was δ-T with a percentage distribution of 13.5–70%. A relatively high amount of δ-T was found in *M.* Bernu prieks apples with a concentration of 21.5 mg mL^−1^. Moreover, this amount was 13.45- and 19.94-fold higher than reported earlier for *Jatropha curcas* L. and *M.* × *domestica* Borkh oil, respectively [9,25].

The first in vivo model assessing the antineoplastic activity of Ts has shown that δ-tocopherol to be the most active homolog compared with α- or γ-T in inhibiting tumor growth, perhaps through trapping reactive oxygen and nitrogen species and inducing apoptosis [30]. The concentration of total Ts varied among the *Malus* species and dessert apples investigated, varying between 16.8 and 30.9 mg mL^−1^. The lowest content of total Ts was found in dessert apple pomace oil, while the highest value for *M.* Bernu prieks, followed by *M*. cv. “Ola”, and *M*. × *Soulardii*. The calculated weight ratios of four Ts (**α-T**:β-T:γ-T:δ-T, average) in three crab and dessert apple pomace oils (excluding *M.* Bernu prieks) were **5.2**:1.6:2.2:1.9 and **4.7**:1.4:0.6:1.8, respectively. Having regard to the high content of δ-T that has been found in the oil of *M*. Bernu prieks; the ratios were calculated individually for this sample, corresponding to 4.4:2.9:.2.1:**21.5**. Only a few studies have previously reported the presence of the Ts as constituents of crab and dessert apple seed oils [9,18]. The authors noted that the seed oils obtained from dessert apple seed oils were characterized by higher contents of Ts (19.1–37.9 mg mL^−1^ oil) when compared to seed oils recovered from crab apples (13.0–20.3 mg mL^−1^). The average ratios of calculated amounts of Ts in crab and dessert apple seed oils were as follows: **4.1**:2.7:1.6:1 and 2.6:2.5:1.1:1 [18]. The results of this study indicate a potential utilization of crab apple pomace as a natural and cheap source of vitamin E. Moreover, the results of this study might be useful for cosmetics companies specializing in the development/production of natural skin-care products.

### 2.3. Phytosterols and Triterpenes of Crab Apple Pomace Oil

Phytosterols are some of the compounds which are distributed among the tissues of plant [31]. A meta-analysis of 41 trials summarized in the excellent review article provided by Katan et al. [32] shows that intake of 2 g/day of stanols or sterols reduced low-density lipoprotein (LDL) by 10%. Therefore, for the last decades, purified phytosterols have been added to numerous food products to enhance the functionality and increase the nutritional value [31]. The phytosterol and triterpene composition of the crab and dessert apple pomace oils analyzed are shown in Table 3.

Eleven biologically active compounds, five phytosterols (campesterol, stigmasterol, β-sitosterol, isofucosterol, and ∆7-avenasterol) and six triterpenes (squalene, α-amyrin, lupeol, 24-methylenecycloartanol, uvaol, and ursolic aldehyde), were successfully identified and quantified in the tested pomace oils. β-Sitosterol was the prevalent compound found in all tested pomace oils with a percentage distribution of 10.3–94.5%. The second dominant compound was campesterol, where the percentage distribution varied in the range 0.1–4.6%. With regard to the *M.* Bernu prieks, stigmasterol was the prevailing phytosterol present in this sample, showing a content of 111.0 mg mL^−1^ oil (87.8%), while in the other oil samples this compound was not found. The results of this study are in agreement with those published previously [9,22,31], where the main phytosterol among the apple oils was β-sitosterol (94.0%, 82.1%, and 97.5%, respectively). However, a minor difference in phytosterols profiles and ratios may be due to the different solvents used for the extraction of phytochemicals, as well as environmental factors and agricultural practices applied during the growing season.

Among the analyzed triterpenes, lupeol and uvaol were found to be dominant compounds of *M.* crab apples, varying in the range of 0.1–66.3% and 0.2–68.9%, respectively. Recent reports indicate the presence of dietary lupeol as a constituent of vegetables and fruit including *Brassica oleracea*, *Capsicum* spp. *Cucumis sativus*, *Solanum lycopersicum*, and of fruits such as *Olea* spp. *Ficus carica*, *Mangifera indica*, *Fragaria* spp. and *Vitis* spp. [33,34], while uvaol in *Prunus avium* [26]. It must be noted, however, that the amounts of lupeol reported here are 99-, 16-, and 2-fold higher than reported earlier for olive fruit, aloe leaves, and ginseng oil, respectively [26]. The recent finding revealed that lupeol with no toxicity is a therapeutic and chemopreventive agent for the treatment of inflammation and cancer [33]. The same statement was proved by Siddique and Saleem [35], pointing out that lupeol is pharmacologically effective in treating various diseases under preclinical settings regardless of the type of administration. The results of this study suggest that the crab apple pomace oils of *Malus* spp., especially *M*. Berzukroga dzeltenais and *M*. cv. “Ola” to be considered as a good source of lupeol which could be incorporated as ingredients in making of high-value functional food products with many of physiological and biochemical effects.

### 2.4. Total Carotenoids of Crab Apple Pomace Oil

While carotenoids are assumed to be present in dessert and crab apple, lemon, grape, mango, melon, and seed oils [31], there have been no reports on quantification specifically of crab apple pomace oils consisting of stems, seeds, flesh, and skin. The results show that the content of carotenoids was found higher in oil recovered from the pomace of yellow crab apple Berzukroga dzeltenais, while the lowest in oil extracted from the pomace of “Bernu prieks” apple, where the concentrations were 14.5 and 5.1 mg mL^−1^, respectively (Table 3). These results are in accordance with those of a previous study investigating different types of palm fruit oils for their carotenoid contents [36], and significantly higher than reported in quince and sunflower seed oils by Fromm et al. [37] Based on a report by Biesalski et al. [38], mean dietary intake required for beta-carotene to benefit from the preventive health potential are estimated to be 2–4 mg/day, thus showing that between ~0.2178 and ~0.4356 mg per day of crab apple pomace oil would ensure the necessary recommended daily dose amount of beta-carotene.

### 2.5. Antioxidant Activity of Crab Apple Pomace Oils

**Figure 3:**
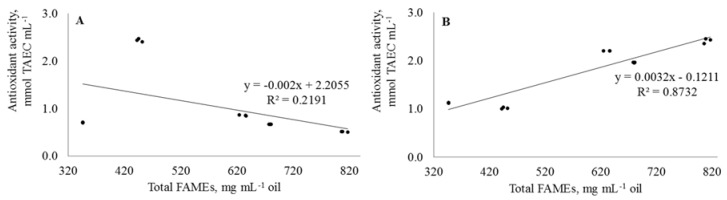
Correlation between total FAMEs (mg mL^−1^ oil) and the antioxidant activities (mmol TAEC mL^−1^) of tested apple pomace oils using: DPPH^•^ assay (**A**); and FRAP assay (**B**).

The antioxidant activity of tested oils of *Malus* apple fruit was studied by radical scavenging capacity using the DPPH^•^ method, while the ascertainment of total antioxidant capacity was done using the FRAP method. The DPPH^•^ radical scavenging capacity of the apple pomace oils shows values ranging between 0.5 and 2.4 mmol TAEC mL^−1^, with *M.* × *soulardii* having higher values, while crab apple *M.* cv. “Ola” having the lowest. Comparable results were reported by Prescha et al. [39] where flax (*Linum usitatissimum*) and rose hip (*Rosa rugosa*) oils were characterized as the strongest contributors to antioxidant activity using the DPPH^•^ radicals. The results of scavenging capacity assay using the DPPH^•^ radicals did not show correlation between the total bound FAs (FAMEs) (R^2^ = 0.2191) (Figure 3A), Ts (R^2^ = 0.0086) or carotenoids (R^2^ = 0.2306) in apple pomace oils. In addition, a strong negative correlation was found between FFAs (silyl derivatives) and DPPH (R^2^ = 0.7206), while a moderate positive correlation was found between FFAs and DPPH values (R^2^ = 0.6581). The opposite results were obtained in a study by Tuberoso et al. [19], where the correlation between the total content of Ts in different commercial oils and scavenging of the DPPH^•^ radical was R^2^ = 0.7000. The data from FRAP assay were significantly different (*p* < 0.05) and no correlation was found with the DPPH^•^. The results showed that the oil recovered from *M.* cv. “Ola” is more active compared with other oils. Generally, the only positive and strong correlation (R^2^ = 0.8732) was found between the bound FAs and FRAP (Figure 3B).

The results of this study show that the different constituents, as well as testing systems, may affect the capacity of oils to quench different radicals. A similar result was reported previously by Wang et al. [40], pointing out that some compounds, which have ABTS radicals scavenging activity, do not show DPPH^•^ activity. Moreover, Radenkovs et al. [41] proposed that radical scavenging (DPPH^•^) with each compound is an independent process, the overall success of which depends predominantly on the chemical structure of a particular biologically active compound, rather than on the concentration.

### 2.6. Antimicrobial Activity of Crab Apple Pomace Oils

MIC is defined as the lowest concentration of an antimicrobial mean that will suppress the visible growth of a microorganism after overnight incubation [42]. In this study, the susceptibility of standard Gram-positive and Gram-negative test bacteria to pomace oils obtained from *Malus* spp. crab apple was estimated in vitro using the method recommended by Balouiri et al. [43]. MIC data are presented in Table 4.

The growth of Gram-positive test bacteria was inhibited only at the highest oil concentrations applied (MIC 125.0 and 61.2 mg mL^−1^) for *M*. × *domestica* Borkh. cv. “Gita”, *M*. × *soulardii*, and *M*. Bernu prieks. However, the pomace oil of *M*. cv. “Ola” had a MIC value of 31.2 mg mL^−1^ for Gram-positive *Staphylococcus aureus.* Different activities of the tested pomace oils towards Gram-negative test bacteria was observed. The pomace oil of *M*. Bernu prieks, *M*. cv. “Ola”, and *M*. Berzukroga dzeltenais exhibited an inhibitory activity with MIC value 31.2 mg mL^−1^ against *E. coli*, while only pomace oil of *M*. cv. “Ola” and *M*. Berzukroga dzeltenais were able to suppress the growth of *P*. *aeruginosa* at a concentration of 31.2 mg mL^−1^. Our data show that tested pomace oils of *M*. spp. had differing antimicrobial activity against bacteria. Only the pomace oil of *M*. cv. “Ola” showed an inhibition of both Gram-positive and Gram-negative test cultures, while, to a lesser extent, the *M*. Berzukroga dzeltenais pomace oil had the ability to inhibit only Gram-negative pathogenic bacteria.

In contrast to our data, some reports have shown significantly better susceptibility of pathogenic bacteria to antimicrobial agents recovered from different plant extracts, however these utilized different inoculum concentrations (in particular 10^5^ CFU mL^−1^) [44]. It was proposed previously that increasing the inoculum concentration from 10^5^ to ≥ 10^8^ CFU mL^−1^ may cause a reduction in bactericidal activity of all the antimicrobial compounds against pathogenic bacteria [45]. The results of this approach show that the growth of pathogenic bacteria probably was influenced by the chemical composition of pomace oils, particularly linoleic and oleic acids [46], which were found to be dominant representatives of pomace oils from *M*. cv. “Ola”, and *M*. Berzukroga dzeltenais. Further in-depth research on evaluation of antimicrobial activity of lipophilic individual isolates from *Malus* spp. is highly recommended.

## 3. Materials and Methods

### 3.1. Plant Material

Crab apple fruit of *Malus* species (*M*. × *soulardii*, *M*. Bernu prieks, *M*. “Ola”, *M*. Berzukroga dzeltenais) were collected in Pūre Horticultural Research Centre Ltd. (Pūre parish, Tukums District, Latvia), GPS location: N: 57°02′23.30″; E: 22°54′48.1″, on 26 September, 2017 (Table S1). Dessert apples of cv. “Gita” (as a reference for comparison) were harvested in Institute of Horticulture (Dobele, Latvia), GPS location: N: 56°36′35.0″; E: 23°17′58.7″ on 13 September 2017 (starch-iodine test—4.8 points from total 10). The ripening stage of “Gita” fruit was assessed using a starch-iodine test [47], while crab apples were harvested at ~121 days after full bloom. The apple trees were cultivated in soils, varying from haplic luvisol (super eutric) to luvisol (hypereutric), while the soil texture, varying from poorly podzolic sandy loam to loamy soil. The integrated growing system and a planting distance of 3 m × 4 m for crab apple trees and 5 m × 3 m for “Gita” apple trees, without irrigation system was used. Grass was mowed several times (~6) during the growing season and the rows were treated with herbicides in the initial part of the vegetation season. Cultivar “Gita” apple trees were grafted on the rootstock B9 and grown in the orchard according to the integrated system using the same conditions. Crab apple fruit were picked from 10 randomly selected eight-year-old trees, while “Gita” fruit were picked from 10 randomly selected seven-year-old trees. About 0.7 ± 0.1 kg of each crab and 1.5 kg of dessert apple fruit were harvested (between 11:00 and 15:00 local time) and transported immediately (within 1 h) to the laboratory of the Institute of Horticulture. All apple fruit were carefully mixed, frozen in a freezer “PORKKA BF 710” (Porkka, Lahti, Finland) at –25 ± 1 °C. Frozen fruits were packed in polypropylene bags (1–1.5 kg in each) and stored in a low-temperature chamber “VTK 201 V” (Holod-MSK, Moscow, Russia) at −18 ± 1 °C until analysis, a maximum of 10 weeks. The juice from apple fruit was obtained (after frozen fruit were thawed to ambient temperature −22 ± 1 °C) using a Voran 60 K basket press at a pressure of 300 bars (Voran Maschinen GmbH, Pichl bei Wels, Austria). Pomace (consisting of stems, seeds, flesh, skin) after juice pressing were gradually freeze-dried using a FreeZone freeze-dry system (Labconco, Kansas City, MO, USA) at −51 ± 1 °C under vacuum of 0.055–0.065 mbar for 20 h, further milled (0.5 mm particle size) using a variable speed rotor mill Pulverisette 14 (Fritsch, Idar-Oberstein, Germany). The moisture content of samples was measured gravimetrically at 103 ± 2 °C using the method of Ruiz [48]. Samples intended for the analysis of fatty acid methyl esters (FAMEs), free fatty acids (FFAs) and phytosterols were packed into Mylar bags (foil Mylar zip-lock bags, thickness 3 mm, New York, NY, USA), frozen (−18 °C) and delivered directly to the laboratory of the University of Latvia, Riga, Latvia.

### 3.2. Chemicals and Standards

Standards of α, β, γ, and δ homologs of tocopherol (T) (purity >95%) were purchased from Merck Millipore (Darmstadt, Germany). Methyl heptadecanoic acid (analytical standard, purity ≥ 99%), γ-linoleic acid (analytical standard, purity ≥ 99%), dodecanal (analytical standard, purity ≥ 98%), 1-octadecanol (ReagentPlus, purity ≥ 99%), n-tetracosane (analytical standard, purity ≥ 99.5%), and ergosterol (pharmaceutical secondary standard, purity 95%) were purchased from Sigma-Aldrich Chemie GmbH (Schnelldorf, Germany), while n-paraffin analytical standard (C_8_–C_40_ alkanes calibration standard) was obtained from Supelco Analytical (Bellefonte, PA, USA). HPLC grade pyridine was purchased from Honeywell Riedel-de Haën GmbH, (Seelze, Germany), HPLC grade methanol, n-hexane, petroleum ether (puriss. p.a., ≥99.9%, boiling point 50–70 °C), bis(trimethylsilyl) trifluoroacetamide (BSTFA) and boron trifluoride–methanol solution (BF_3_/MeOH) were purchased from Sigma-Aldrich Chemie Ltd., (St. Louis, MO, USA). The 2.2-diphenyl-1-picrylhydrazyl, 2-propanol (HPLC grade), resazurin sodium salt were obtained from Sigma-Aldrich Chemie GmbH (Steinheim, Germany). Deionized water was prepared using an Elix Advantage 3 water purification system (Millipore S.A.S., Molsheim, France).

### 3.3. Soxhlet Extraction

Soxhlet extraction was done using the method of Abdolshahi et al. [49] with slight modification. Triplicate samples about of 10 g of freeze-dried and finely ground pomace were accurately weighted in extraction thimbles (Whatman single thickness, 33 × 94 mm). Further, the thimbles placed inside the extraction chambers and submitted to a Soxhlet extraction using the system B-811 (BÜCHI Labortechnik AG, Flawil, Switzerland), which is fully automated. The samples were extracted using 100 mL of petroleum ether for 3 h. Sufficient heat (Soxhlet warm extraction at 70 °C) was used to give about 10 cycles of solvent per h. At the end of the extraction, oil samples were dried to release the solvent from oil extracts (using the above-mentioned Soxhlet system), further Soxhlet beakers were placed in a desiccator to cool and weighted to determine the yield of crude oil. Oil samples were transferred to 15 mL plastic tubes (Sarstedt AG & Co. KG, Nümbrecht, Germany) and stored at −18 ± 1 °C until analysis, a maximum of two weeks.

### 3.4. Preparation of FAs for GC/MS Analysis

Methylation of polyfunctional compounds in oil samples was carried out using the method of Lelacheur et al. [50] with slight modification. Briefly, 0.25 µL of 14% BF_3_/MeOH was added to each 0.1 mL oil sample in 22 mL glass vials (PerkinElmer, Waltham, MA, USA) and heated for a minimum of 1 h at 70 °C. Tubes were then allowed to cool for approximately 20 min. When tubes reached ambient temperature, 3.0 mL of double distilled water (DDW) and 3.0 mL of n-hexane were added to each tube followed by vortex-mixing for 15 s. After centrifugation at 3200× *g* for 4 min, the upper n-hexane layer was quantitatively transferred to a 15 mL plastic tube. Each sample was extracted thrice using the above-mentioned procedure. The supernatant fractions were hereafter flushed with nitrogen for ~5 min and dry residues stored in a low-temperature chamber “VTK 201 V” (Holod-MSK, Moscow, Russia) at −18 ± 1 °C until analysis, a maximum of two weeks. Directly prior to GC/MS analysis, a 0.1 g of each oil sample (FAMEs) was reconstituted with 1 mL of pyridine.

### 3.5. GC Conditions for FAMEs

The analysis of FAMEs was carried out on a Clarus 580 system PerkinElmer, Inc. (Waltham, MA, USA) equipped with a quadrupole analyzer Clarus SQ 8 C mass-selective detector (Waltham, MA, USA). All analyses of FAMEs were done using Omegawax 250 (Sigma-Aldrich Chemie GmbH, Taufkirchen, Germany) column with a stationary phase of intermediate polarity (30 m × 0.25 mm, sorbent thickness—0.25 μm). The injector temperature has been set to +280 °C; automatic injection using an autosampler, injection volume 0.5 μL; split ration 4:1. The initial oven temperature was maintained at 75 °C for 2 min, then raised to 150 °C (rate of 20 °C min^−1^), then increased to 270 °C (rate of 4 °C min^−1^). Helium (ultra-high purity 5.0 grade—99.999%) was used as a carrier gas at the initial flow rate of 2.0 min^−1^ and then held constant at 1.0 min^−1^ with the split ratio 4:1. The total separation time was 35.75 min.

### 3.6. GC Conditions for FFAs, Phytosterols, and Triterpenes

After silylation using the method of Lelacheur et al. [50], oils were analyzed using the above-mentioned GC system. All analyses of the silylated derivatives were done using an Elite-5MS capillary column (30 m × 0.25 mm i.d. and film thickness of 0.25 μm, PerkinElmer, Waltham, MA, USA). The injector temperature was set to +290 °C; automatic injection using an autosampler, injection volume 1.0 μL; split ratio 4:1. The initial oven temperature was maintained at 75 °C for 2 min, then raised to 130 °C (rate of 20 °C min^−1^), and after at a rate of 4 °C min^−1^ increased to 310 °C, followed by an isothermal operation for 5 min. Helium (ultra-high purity 5.0 grade—99.999%) was used as a carrier gas at the initial flow rate of 2.0 min^−1^ for 0.5 min^−1^, and then followed by a constant flow at 1.0 min^−1^. The total separation time was 54.75 min.

### 3.7. The MS Conditions for FAMEs and Silyl Derivatives Detection

Detector mode: Electron impact ionization was at 70 eV; ion source temperature: +230 °C; inlet temperature was +290 and 280 °C for silyl and FAMEs derivatives, respectively; capture time for FAMEs starting from 3.5 min (1.7 scan s^−1^), while from 0.5 min (0.50 scan s^−1^) for silyl derivatives; ion multiplier: 1700 V; and ion *m*/*z* interval: 42–550 for FAMEs and 35–750 for silyl derivatives.

### 3.8. Standard Compounds Used for Calibration

Methyl heptadecanoic acid for FAMEs, γ-linoleic acid for FFAs and glycidyl fatty acid esters, 1-octadecanol for aliphatic alcohols, dodecanal for aldehydes, n-tetracosane for alkanes, and ergosterol for phytosterols and triterpenes. Least squares regression analysis was implemented, using the peak area ratios against increasing standard concentrations to obtain calibration linearity (y = ax + b, where b is gradient of the slope and a is intercept point at *y*-axis (i.e., when x = 0) (Table S2). The precision of the method was assessed by triplicate analysis of standard solutions at five concentrations.

Retention indexes (RI) for temperature programmed GC analysis at a constant heating rate were calculated using the method of van Den Dool and Kratz [51] based on n-paraffin (C_8_–C_40_) standard mixture. The components were identified by comparison of their RI and mass spectral data using an in-house built library and the NIST mass spectral library NIST MS 2.2.

### 3.9. Tocopherol Determination Using the RP-HPLC/FLD

Tocopherols (Ts) were determined using the previously validated method of Górnaś et al. [52]. Crab and dessert apple pomace oils (0.1 g) were diluted in 2-propanol to a volume of 10 mL and filtered through an MS^®^ nylon syringe filter with 0.22 µm pore size (Membrane Solutions, Plano, TX, USA) into 1.5 mL glass vials and immediately analyzed using the HPLC system. The chromatographic separation was carried out using the Shimadzu HPLC system (Shimadzu Corporation, Kyoto, Japan) consisting of a pump (LC-10ADvp), a degasser (DGU-14A), a low-pressure gradient unit (FCV-10ALvp), a system controller (SCL-10Avp), an auto injector (SIL-10AF), a column oven (CTO-10ASvp), a fluorescence detector (RF-10AXL) and a Luna PFP column (3 µm, 150 × 4.6 mm) with a guard column (4 mm × 3 mm) (Phenomenex, Torrance, CA, USA). The analysis was done using the following conditions: mobile phase methanol:water (93:7; *v*/*v*); flow (1.0 mL min^−1^); and column oven temperature (40 °C). The total separation time was 13.0 min. The identification and quantification of each of tocochromanols homolog was estimated using a fluorescence detector at an excitation wavelength of 295 nm and emission wavelength of 330 nm.

### 3.10. Spectrophotometric Analysis of the Total Carotenoids

**Figure 4:**
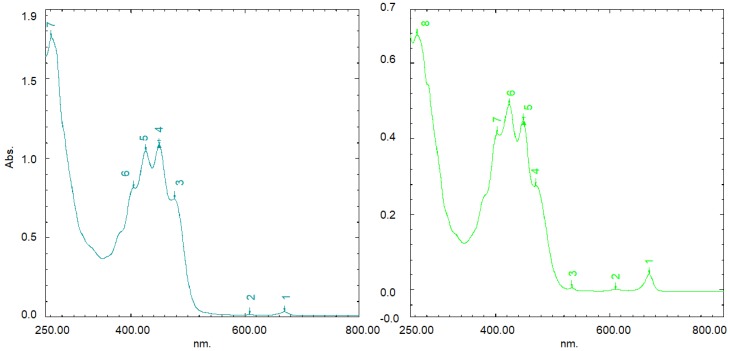
The typical UV-VIS spectrum of carotenoid interference between 390 and 480 nm.

Total carotenoids were estimated using the method of Biehler et al. [53] As reported, the spectrophotometric method (method of mean) is robust with high reproducibility, sensitivity, and strong correlation with the HPLC method. The method is based on the mean absorption coefficients and mean absorption wavelength (Figure 4).

Humans typically consume a wide variety of carotenoids in the diet; β-carotene, along with lycopene, lutein, zeaxanthin, β-cryptoxanthin, and α-carotene, account for 90% of circulating carotenoids [54]. Therefore, considering exclusively these major abundant carotenoids, an average absorption coefficient is equivalent to ε = 135,310.

To estimate the average carotenoid concentrations (mol L^−1^), the following equation was used:

(1) $$\;c\left({\mathrm{mol}\;L}^{-1}\right)=\;\frac{A_{450\;\times }\;F_{d}}{135,310}$$

with A_450_ being the mean absorbance maximum (A450), d = 1 cm, and F a dilution factor adjusting for extractions, drying, and reconstitution processes. Using an average molar mass (g mol^−1^), results can also be expressed as g per L^−1^ and as mg per 100 g^−1^.

A 0.1 g oil sample was 10 times diluted using petroleum ether in centrifuge tubes (15 mL), and the absorbance was measured immediately at 450 nm using a UV-1800—Visible Spectrophotometer SHIMADZU (Shimadzu Corp., Kyoto, Japan). Results were expressed as mg β-carotene equivalent per mL^−1^ (mg mL^−1^).

### 3.11. Antioxidant Assays

#### 3.11.1. DPPH^•^ Radical Scavenging Activity

The radical scavenging activity, using the free-radical DPPH^•^ assay was determined using the method of Li et al. [55] with slight modification. Briefly, the oils (100 μL) were reacted with 2.9 mL of DPPH^•^ solution (0.0039 g DPPH^•^ in 100 mL methanol). Absorbance of the oil extracts was done at 515 nm using a spectrophotometer. The absorbance results were converted using a calibration curve of the standard and expressed as mmol Trolox equivalent antioxidant capacity per mL^−1^ of oil (mmol TEAC mL^−1^).

#### 3.11.2. The Ferric Reducing Antioxidant Power (FRAP) Assay

Free reducing antioxidant power (FRAP) was determined using the method of Radenkovs et al. [41]. The FRAP reagent was prepared daily from 300 mL sodium acetate buffer (0.3 mol L^−1^; pH 3.6), 2,4,6-tripyridyl-s-triazine (TPTZ) solution in 40 mmol L^−1^ of HCl and FeCl_3_ (20 mmol L^−1^). The three solutions were mixed together at the ratio of 10:1:1 (*v*/*v*/*v*), respectively, and then warmed to 37 °C. Extracts and standard (FeSO_4_·7H_2_O) or double distilled water (DDW) for blanks (100 µL) were mixed with FRAP reagent (3.6 mL). The change in absorbance from red to blue was followed at 593 nm after 10 min. A Trolox calibration curve was done between 0.1 and 1.0 mg mL^−1^. The absorbance results were converted using a calibration curve of the standard and expressed as mmol Trolox equivalent antioxidant capacity per mL^−1^ of oil (mmol TEAC mL^−1^).

### 3.12. Minimum Inhibitory Concentration (MIC) Determination

The minimum inhibitory concentration (MIC) was determined using the microdilution method in 96-well plates [43]. Five standard strains of test microorganisms were used for antimicrobial sensitivity testing included Gram-positive and Gram-negative bacteria (*Pseudomonas aeruginosa* ATCC10145; *E. coli* ATCC25922; *Enterococcus faecalis* ATCC29212; *Streptococcus pyogenes* ATCC19615; and *Staphylococcus aureus* ATCC65388/NCTC7447). Bacteria were grown on Nutrient agar (NA, Oxoid, CM0003, ThermoFisher Scientific, Hampshire, UK) at 37 °C for 20–24 h.

Prior to analysis, the microorganism colonies were suspended in Mueller Hilton broth (MHB, Ref. 4017412, Biolife, Milano, Italy) and the suspension was adjusted using the DEN-1B McFarland Tube Densitometer (Grant Instruments Ltd., Cambridge, United Kingdom), to the final turbidity of 0.5 McFarland units which corresponds to 10^8^ CFU mL^−1^ [43]. Under aseptic conditions, the tested oils were solubilized in 50% (*v*/*v*) of dimethyl sulfoxide (DMSO) (Merck Millipore, Darmstadt, Germany) in DDW in the range from 0.9 to 125.0 mg mL^−1^. The negative control consisted of DMSO, MHB, and test cultures. For the broth microdilution test, 100 µL of each bacterial suspension in broth medium was added in each well already containing 100 µL of two-fold serially diluted plant extract. The final volume in each well was 300 µL. Viability of the bacterial cell was visualized using 30 μL/well of 0.01% resazurin aqueous solution. The plates were incubated at 37 ± 1 °C for 24 h. The MIC values were identified as the lowest concentration in which no viable bacterial were observed.

### 3.13. Statistical Analysis

The results obtained are shown as means ± standard error of the mean from three replicates (*n* = 3). The *p*-value < 0.05 was used to denote significant differences between mean values determined using one-way analysis of variance (ANOVA) and the Duncan’s multiple range test done using the assistance of IBM^®^ SPSS^®^ Statistics program 20.0 (SPSS Inc., Chicago, IL, USA).

## 4. Conclusions

The majority of the identified fatty acids in the oils recovered from by-products of *Malus* spp. were found in bound forms, in the form of di- and triglycerides, and fatty acid alkyl esters (from 446.5 to 810.4 mg mL^−1^). Only small amounts of less stable free fatty acids were found, varying in the range of 3.0–26.5 mg mL^−1^. The oils recovered from by-products of *Malus* spp., especially *M*. Bernu prieks, followed by *M*. cv. “Ola”, and *M*. × *Soulardii* are rich in Ts. A relatively high amount of δ-T was found in *M.* Bernu prieks apples with a concentration of 21.5 mg mL^−1^. This amount was 13.45- and 19.94-fold higher than reported earlier for *Jatropha curcas* L. and *M*. × *domestica* Borkh oil, respectively. In total, 11 compounds including five phytosterols and six triterpenes were identified and quantified in the tested pomace oils. β-Sitosterol was the prevalent compound found in all tested pomace oils with a percentage distribution of 10.3–94.5%. Among the analyzed triterpenes, lupeol was found to be the dominant compounds in *M.* crab apples, varying in the range of 0.1–66.3%. Thus far, this is the first report providing the evidence on the presence of dietary lupeol in the *M*. crab apples. Moreover, the amount of lupeol reported here are 99-, 16-, and 2-fold higher than observed earlier for olive fruit, aloe leaves, and ginseng oil, respectively. The concentration of total carotenoids was found to be higher in oil recovered from the pomace of yellow crab apple Berzukroga dzeltenais, while the lowest in oil extracted from the pomace of Bernu prieks apple, corresponding to 14.5 and 5.1 mg mL^−1^, respectively. The results of scavenging capacity assay using the DPPH^•^ radicals did not show correlation between the total bound FAs (R^2^ = 0.2191), tocopherols (R^2^ = 0.0086) or carotenoids (R^2^ = 0.2306) in apple pomace oils, which suggests that radical scavenging (DPPH^•^) with each compound is an independent process, the overall success of which depends predominantly on the chemical structure of a particular biologically active compound, rather than on the concentration. The only pomace oil from *M*. cv. “Ola” showed an inhibition of both Gram-positive and Gram-negative test cultures, while, to a lesser extent, the ability to inhibit only Gram-negative pathogenic bacteria was attributed to pomace oil from *M*. Berzukroga dzeltenais.

## Figures and Tables

**Table 1:** Content of fatty acids (FAME derivatives) and tocopherols (Ts) in pomace oils recovered from *Malus* spp. crab apples, mg mL^−1^.

Compound	Retention Index	“Gita”	*Malus soulardii*	Bernu Prieks	“Ola”	Berzukroga Dzeltenais
**FAs**						
Dodecanoic acid	1758	n.d.	7.2 ± 0.0 ^a^	7.5 ± 0.0 ^a^	3.6 ± 0.0 ^c^	5.3 ± 0.1 ^b^
Tetradecanoic acid	1965	9.2 ± 0.0 ^a^	8.2 ± 0.1 ^b^	8.1 ± 0.1 ^b^	3.9 ± 0.0 ^d^	6.0 ± 0.0 ^c^
9-Oxononanoic acid	2016	n.d.	n.d.	n.d.	4.9 ± 0.0 ^a^	5.6 ± 0.0 ^a^
9,9-Dimethoxynonanoic acid	2050	n.d.	7.3 ± 0.1 ^b^	8.9 ± 0.0 ^a^	5.1 ± 0.0 ^c^	4.6 ± 0.0 ^c^
Nonanedioic acid	2092	9.6 ± 0.0 ^a^	8.3 ± 0.1 ^b^	9.1 ± 0.0 ^ab^	n.d.	n.d.
Hexadecanoic acid	2165	44.8 ± 0.2 ^d^	56.8 ± 1.6 ^c^	71.3 ± 1.3 ^b^	70.9 ± 1.7 ^b^	81.5 ± 2.7 ^a^
(Z)-7-Hexadecenoic acid	2186	n.d.	7.1 ± 0.2	n.d.	n.d.	n.d.
(Z)-9-Hexadecenoic acid	2192	9.5 ± 0.1 ^a^	7.2 ± 0.2 ^b^	7.9 ± 0.4 ^b^	n.d.	n.d.
Heptadecanoic acid	2261	n.d.	n.d.	n.d.	3.6 ± 0.0 ^b^	4.7 ± 0.1 ^a^
Octadecanoic acid	2367	18.6 ± 0.1 ^b^	16.3 ± 0.1 ^c^	22.2 ± 0.3 ^a^	14.4 ± 0.2 ^d^	9.2 ± 0.1 ^e^
(Z)-9-Octadecenoic acid	2385	67.1 ± 0.3 ^e^	95.8 ± 0.4 ^d^	136.0 ± 2.1 ^c^	206.9 ± 1.8 ^a^	164.1 ± 2.1 ^b^
9,10-Dihydroxystearate	2395	1.5 ± 0.0 ^ac^	1.6 ± 0.1 ^ac^	1.1 ± 0.0 ^ab^	0.8 ± 0.0 ^b^	1.0 ± 0.0 ^bc^
(Z,Z)- 9,12-Octadecadienoic acid	2433	149.0 ± 0.2 ^e^	193.5 ± 4.0 ^d^	307.8 ± 4.6 ^c^	468.8 ± 3.1 ^a^	377.9 ± 2.2 ^b^
(Z,Z,Z)-9,12,15-Octadecatrienoic acid	2496	14.6 ± 0.1 ^c^	19.4 ± 0.4 ^b^	29.5 ± 0.6 ^a^	14.6 ± 0.3 ^c^	9.9 ± 0.0 ^d^
Eicosanoic acid	2571	13.7 ± 0.1 ^a^	9.6 ± 0.0 ^b^	14.4 ± 0.2 ^a^	7.9 ± 0.1 ^c^	5.8 ± 0.0 ^d^
(Z)-11-Eicosenoic acid	2588	10.2 ± 0.1	n.d.	n.d.	n.d.	n.d.
Docosanoic acid	2781	n.d.	9.7 ± 0.0 ^a^	9.6 ± 0.0 ^a^	5.8 ± 0.0 ^b^	5.0 ± 0.0 ^b^
**Total FAs**		**346.2 ± 1.2 ^e^**	**446.5 ± 7.3 ^d^**	**632.3 ± 9.6 ^c^**	**810.4 ± 7.2 ^a^**	**679.6 ± 7.3 ^b^**
**Alcohol**						
Nonacosan-10-ol	3046	6.6 ± 0.1 ^b^	16.7 ± 0.2 ^a^	4.9 ± 0.0 ^c^	3.9 ± 0.1 ^d^	3.2 ± 0.0 ^d^
**Ts**						
δ-Tocopherol	8.1	3.6± 0.1 ^c^	3.1 ± 0.1 ^c^	21.5 ± 0.3 ^a^	4.8 ± 0.1 ^b^	3.6 ± 0.1 ^c^
β-Tocopherol	9.8	2.7 ± 0.1 ^b^	3.9 ± 0.1 ^a^	2.9 ± 0.1 ^b^	2.6 ± 0.1 ^b^	3.0 ± 0.1 ^b^
γ-Tocopherol	10.3	1.1 ± 0.1 ^d^	2.3 ± 0.1 ^c^	2.1 ± 0.1 ^c^	6.8 ± 0.1 ^a^	4.4 ± 0.1 ^b^
α-Tocopherol	12.0	9.4 ± 0.2 ^c^	13.9 ± 0.3 ^a^	4.4 ± 0.3 ^e^	10.2 ± 0.2 ^b^	7.4 ± 0.2 ^d^
**Total Ts**		**16.8 ± 0.5 ^f^**	**23.1 ± 0.6 ^c^**	**30.9 ± 0.8 ^a^**	**24.4 ± 0.5 ^d^**	**18.4 ± 0.5 ^e^**

Note: All measurements were done in triplicate (*n* = 3). Values with different superscripts within the same indices are significantly different (*p* < 0.05), one-way repeated ANOVA and Duncan’s multiple range test. n.d., not detected; for tocopherols, only retention times (min.) are given in the table.

**Table 2:** Content of free fatty acids, glycidyl fatty acids, aliphatic alcohols, aldehydes, and alkanes in pomace oils recovered from *Malus* spp. crab apples, mg mL^−1^.

Compound	Retention index	“Gita”	*Malus soulardii*	Bernu Prieks	“Ola”	Berzukroga Dzeltenais
**FFAs**						
Pentadecanoic acid	1254	0.01 ± 0.0 ^b^	0.1 ± 0.0 ^a^	0.03 ± 0.0 ^b^	0.01 ± 0.0 ^b^	0.04 ± 0.0 ^b^
Hexadecanoic acid	2045	3.8 ± 0.1 ^a^	1.2 ± 0.0 ^c^	3.2 ± 0.1 ^ab^	2.6 ± 0.1 ^b^	3.0 ± 0.0 ^ab^
(Z,Z)-9,12-Octadecadienoic acid	2206	2.6 ± 0.1 ^d^	0.4 ± 0.0 ^e^	4.0 ± 0.0 ^c^	14.4 ± 0.1 ^a^	7.1 ± 0.0 ^b^
(Z)-9-Octadecenoic acid	2212	4.2 ± 0.0 ^c^	0.8 ± 0.0 ^d^	3.8 ± 0.0 ^c^	8.3 ± 0.1 ^a^	5.8 ± 0.1 ^b^
(E)-9-Octadecenoic acid	2220	1.1 ± 0.0 ^a^	0.3 ± 0.0 ^b^	1.1 ± 0.0 ^a^	0.5 ± 0.0 ^b^	0.7 ± 0.0 ^b^
Octadecanoic acid	2239	1.3 ± 0.0 ^ab^	0.3 ± 0.0 ^c^	1.6 ± 0.0 ^a^	0.7 ± 0.0 ^b c^	1.0 ± 0.1 ^ab^
**Total FFAs**		**13.1 ± 0.2 ^c^**	**3.0 ± 0.0 ^d^**	**13.6 ± 0.1 ^c^**	**26.5 ± 0.3 ^a^**	**17.6 ± 0.0 ^b^**
**Glycidyl fatty acid esters, glycerides (GFAE)**						
1,2,4-Butanetriol	1387	8.5 ± 0.1 ^a^	0.1 ± 0.0 ^b^	n.d.	n.d.	n.d.
1-Monopalmitoylglycerol	2576	n.d.	n.d.	n.d.	0.2 ± 0.0 ^a^	0.01 ± 0.00 ^b^
2-Monolinoleoylglycerol	2703	n.d.	n.d.	n.d.	0.04 ± 0.01	n.d.
1-Monolinoleoylglycerol	2732	0.4 ± 0.0 ^b^	0.3 ± 0.0 ^b^	0.3 ± 0.0 ^b^	2.0 ± 0.0 ^a^	0.3± 0.0 ^b^
1-Monooleoylglycerol	2740	n.d.	n.d.	n.d.	0.56 ± 0.0	n.d.
2-Monostearoylglycerol	2766	n.d.	n.d.	n.d.	0.06 ± 0.0	n.d.
**Total GFAE**		**8.9 ± 0.1 ^a^**	**0.4 ± 0.0c**	**0.3 ± 0.0c**	**2.9 ± 0.1 ^b^**	**0.3 ± 0.0 ^c^**
**Aliphatic alcohols**						
1-Octadecanol	2155	2.6 ± 0.0 ^a^	0.9 ± 0.0 ^b^	1.4 ± 0.0 ^b^	n.d.	n.d.
1-Hexacosanol	2930	3.7 ± 0.0 ^a^	1.7 ± 0.0 ^b^	1.8 ± 0.0 ^b^	0.8 ± 0.0 ^c^	2.0 ± 0.0 ^b^
Nonacosan-10-ol	3046	47.8 ± 0.7 ^a^	37.6 ± 1.5 ^b^	30.5 ± 0.4 ^c^	14.5 ± 0.0 ^d^	14.2 ± 0.1 ^d^
1-Octacosanol	3127	3.5 ± 0.0 ^a^	1.6 ± 0.0 ^b^	1.9 ± 0.0 ^b^	0.8 ± 0.0 ^c^	1.8 ± 0.0 ^b^
**Total aliphatic alcohols**		**57.5 ± 0.7 ^a^**	**41.8 ± 1.5 ^b^**	**35.6 ± 0.4 ^c^**	**16.1 ± 0.0 ^e^**	**18.0 ± 0.1 ^d^**
**Alkanes**						
Pentacosane	2500	2.4 ± 0.1	n.d.	n.d.	n.d.	n.d.
Heptacosane	2700	8.2 ± 0.1 ^a^	1.3 ± 0.1 ^c^	2.5 ± 0.0 ^b^	0.81 ± 0.0 ^d^	1.5 ± 0.0 ^c^
Octacosane	2800	2.8 ± 0.0 ^a^	1.1 ± 0.0b ^c^	1.6 ± 0.0 ^b^	0.7 ± 0.0 ^c^	1.3 ± 0.0 ^bc^
Nonacosane	2900	60.6± 0.9 ^b^	52.1 ± 0.6 ^c^	61.8 ± 0.3 ^a^	28.7 ± 0.1 ^d^	14.9 ± 0.1 ^e^
Triacontane	3000	n.d.	n.d.	n.d.	0.6 ± 0.0	n.d.
Hentriacontane	3100	n.d.	n.d.	n.d.	0.7 ± 0.0	n.d.
**Total alkanes**		**74.1 ± 1.1 ^a^**	**54.6 ± 0.7 ^c^**	**65.9 ± 0.3 ^b^**	**31.6 ± 0.1 ^d^**	**17.7 ± 0.1 ^e^**
**Aldehydes**						
Triacontanal	3254	n.d.	n.d.	n.d.	4.27 ± 0.0 ^b^	8.95 ± 0.0 ^a^

Note: All measurements were done in triplicate (*n* = 3). Values with different superscripts within the same indices are significantly different (*p* < 0.05). n.d., not detected.

**Table 3:** Content of phytosterols and triterpenes in pomace oils recovered from *Malus* spp. crab apples, mg mL^−1^.

Compound	Retention Index	“Gita”	*Malus soulardii*	Bernu Prieks	“Ola”	Berzukroga Dzeltenais
**Phytosterols**						
Campesterol	3230	1.4 ± 0.0 ^b^	2.5 ± 0.6 ^a^	0.1 ± 0.0 ^c^	0.8 ± 0.0 ^c^	1.7 ± 0.0 ^b^
Stigmasterol	3271	n.d.	n.d.	111.0 ± 0.8 ^a^	0.01 ± 0.0 ^b^	n.d.
β-Sitosterol	3341	73.2 ± 0.5 ^a^	51.5 ± 0.9 ^c^	13.1 ± 0.7 ^e^	33.2 ± 0.8 ^d^	52.8 ± 0.6 ^b^
Isofucosterol	3350	2.8 ± 0.0 ^a^	0.6 ± 0.0 ^c^	n.d.	1.6 ± 0.0 ^b^	1.3 ± 0.1 ^b^
∆7-Avenasterol	3394	n.d.	n.d.	0.7 ± 0.0	n.d.	n.d.
**Total sterols**		**77.4 ± 0.5 ^b^**	**54.6 ± 1.5 ^d^**	**126.1 ± 1.5 ^a^**	**35.6 ± 0.8 ^e^**	**55.8 ± 0.7 ^c^**
**Triterpenes**						
Squalene	2794	n.d.	n.d.	2.1± 0.0 ^a^	0.6 ± 0.0 ^b^	1.3 ± 0.0 ^b^
α-Amyrin	3378	n.d.	0.5 ± 0.1 ^b^	2.5 ± 0.1 ^a^	n.d.	n.d.
Lupeol	3384	2.1 ± 0.1 ^b^	0.05 ± 0.0 ^e^	0.2 ± 0.0 ^d^	1.3 ± 0.1 ^c^	3.0 ± 0.1 ^a^
24-Methylenecycloartanol	3437	n.d.	n.d.	1.4 ± 0.0	n.d.	n.d.
Uvaol	3510	6.6 ± 0.3 ^a^	0.1 ± 0.0 ^b^	0.7 ± 0.0 ^b^	0.2 ± 0.0 ^b^	0.2 ± 0.0 ^b^
Ursolic aldehyde	3605	0.9 ± 0.1 ^b^	n.d.	2.1 ± 0.0 ^a^	0.1 ± 0.0 ^b^	0.02 ± 0.0 ^c^
**Total triterpenes**		**9.6 ± 0.4 ^a^**	**0.7 ± 0.0 ^e^**	**5.3 ± 0.1 ^b^**	**2.2 ± 0.1 ^d^**	**4.5 ± 0.1 ^c^**
**Total carotenoids**		6.4 ± 0.5 ^d^	12.8 ± 0.7 ^b^	5.1 ± 0.5 ^c^	5.4 ± 0.5 ^c^	14.5 ± 0.6 ^a^
**DPPH^•^ assay, mmol TAEC mL^−1^**		0.7 ± 0.0 ^c^	2.4 ± 0.4 ^a^	0.8 ± 0.1 ^b^	0.5 ± 0.0 ^d^	0.7 ± 0.0 ^c^
**FRAP assay, mmol TAEC mL^−1^**		1.1 ± 0.4 ^d^	1.0 ± 0.2 ^d^	2.2 ± 0.2 ^b^	2.4 ± 0.2 ^a^	1.9 ± 0.3 ^c^

Note: All measurements were done in triplicate (*n* = 3). Values with different superscripts within the same indices are significantly different (*p* < 0.05). n.d., not detected.

**Table 4:** Minimal inhibitory concentration values of the five *Malus* spp. pomace oils against five reference test cultures, mg mL^−1^ *.

Species	“Gita”	*Malus soulardii*	Bernu Prieks	“Ola”	Berzukroga Dzeltenais
*Staphylococcus aureus*	62.5	62.5	62.5	**31.2**	62.5
*Streptococcus pyogenes*	62.5	62.5	62.5	62.5	62.5
*Enterococcus faecalis*	62.5	62.5	62.5	62.5	62.5
*Pseudomonas aeruginosa*	62.5	62.5	62.5	**31.2**	**31.2**
*Escherichia coli*	62.5	62.5	**31.2**	**31.2**	**31.2**

* Gentamicin was employed as positive control. The MIC to gentamicin was 0.004 mg mL^–1^ in all experiments.

## Supplementary Materials

The following are available online at http://www.mdpi.com/2223-7747/7/4/90/s1, Table S1: The list of fruit used in this study; Table S2: Calibration data for the standards.
