# Supplementary material for: Valorization of Wild Apple (Malus spp.) By-Products as a Source of Essential Fatty Acids, Tocopherols and Phytosterols with Antimicrobial Activity
Source: Plants (Basel). 2018 Oct 24;7(4):90. doi: 10.3390/plants7040090 (PMC6313870; doi:10.3390/plants7040090)
Supplement: Supplementary file 1 [file plants-07-00090-s001.pdf]

**Supplementary Materials**

**Table 1S.** The list of fruit used in this study.

| Latin name | Image | Source | Origin | Main features | Use |
|---|---|---|---|---|---|
| *M.* × *soulardii* | 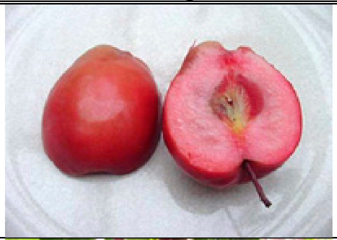 | Received in 2010 within the framework of mutual cooperation from the Gottingen Botanical Garden. | A hybrid of garden origin, probably *M. ioensis* × *M. pumila* | The fruits are medium to small, mostly oval in shape, with a dark pink/red skin and flesh color. Crunchy, juicy. Scab-resistant. Excellent winter hardiness. High productivity. Dark red skin and flesh color. | For wine and cider, sauce, souffle and marshmallow production. |
| *M.* Bernu prieks | 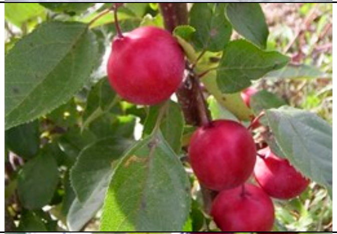 | Found in 2002 at Kandava, Ozolaji, in the garden of the Latvian breeder Rudolf Aker. | Most likely this is Budagovsky's open-pollinated seedling | The fruits are small in size, 20-30 g, mostly pear-shaped. Red flowers, dark red skin and red-pink flesh. Ripen early. A distinctive lingonberry aroma. Excellent winter hardiness. High productivity. | For juice, jam, wine and cider production. |
| *M.* sp., cv. 'Ola' | 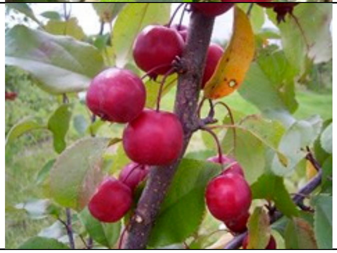 | The cultivar was bred by the Polish breeder Jan Grąbczewski before 1990. | Cultivar was probably derived from the pink apple *M.* × *purpurea* | The fruits are small in size 15-25 g, mostly round-shaped. Dark red skin and flesh color. A distinctive sourness and almond aroma, without astringency. Scab-resistant. Excellent winter hardiness. High productivity. | For jam, stewed fruit production |

**Continuation of Table 1**

| Latin name | Image | Source | Origin | Main features | Use |
|---|---|---|---|---|---|
| *M.* Berzukroga dzeltenais | 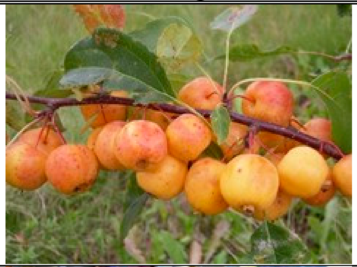 | Found in 2002 along the roadside in Cesis, Latvia. The mother tree has been removed. | Accidental seedling of unknown origin. | Fruits small in size - 15-20 g, round-shaped with a yellow skin and flesh, crisp, juice, with a very distinctive apple-pineapple flavor that is perfectly preserved in wines and ciders even in large dilutions. | For juice, wine and cider production. |
| *M.* × *domestica* Borkh. cv. 'Gita' | 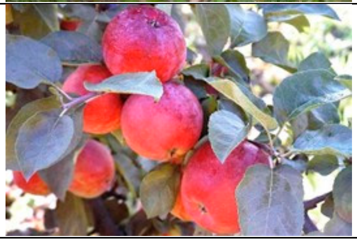 | Latvian high-quality cultivar of domesticated apple which was developed at the Institute of Horticulture in 2009. Breeder – Laila Ikase. | Cross of Liberty' × 'Melba'. | Fruits are large, with red blush and stripes, sub-acid, juicy and crisp. Scab-resistant with rather good tolerance to mildew and other diseases. The tree is very productive with regular yield.s | For fresh consumption and processing. |

**Table 2S.** Calibration data for the standards.

| Standard | Linearity range, mg mL$^{-1}$ | a (mean ± SD) | b (mean ± SD) | $r^2$ |
|---|---|---|---|---|
| Methyl heptadecanoic acid for methyl esters | 0.133 – 1.133 | $5.65 \times 10^6 \pm 9.252 \times 10^6$ | $2.70 \times 10^6 \pm 0.232 \times 10^6$ | 0.9990 |
| γ-Linoleic acid for silyl derivatives | 0.100 – 0.900 | $9.14 \times 10^5 \pm 16.252 \times 10^5$ | $7.545 \times 10^8 \pm 0.052 \times 10^8$ | 0.9995 |
| Dodecanal for silyl derivatives | 0.040 – 0.500 | $-1.82 \times 10^7 \pm 22.34 \times 10^7$ | $6.386 \times 10^8 \pm 0.971 \times 10^8$ | 0.9995 |
| Ergosterol for silyl derivatives | 0.100 – 0.900 | $6.26 \times 10^5 \pm 7.16 \times 10^5$ | $3.581 \times 10^8 \pm 0.024 \times 10^8$ | 0.9994 |
| Octadecanol for silyl derivatives | 0.008 – 0.160 | $-3.42 \times 10^6 \pm 0.23 \times 10^6$ | $8.555 \times 10^8 \pm 0.042 \times 10^8$ | 0.9998 |
| n-Tetracosane for silyl derivatives | 0.008 – 0.160 | $-2.71 \times 10^6 \pm 0.31 \times 10^6$ | $7.722 \times 10^8 \pm 0.050 \times 10^8$ | 0.9996 |
